# A Comparison of Global Gene Expression Measurement Technologies in *Arabidopsis thaliana*

**DOI:** 10.1002/cfg.397

**Published:** 2004-04

**Authors:** Sean J. Coughlan, Vikas Agrawal, Blake Meyers

**Affiliations:** 1 Agilent Technologies Inc. Little Falls Site 2850 Centerville Road Wilmington DE 19808- 1644 USA; 2 Department of Plant and Soil Sciences and Delaware Biotechnology Institute University of Delaware 15 Innovation Way Newark DE 19711 USA

## Abstract

Microarrays and tag-based transcriptional profiling technologies represent diverse but complementary data types. We are currently conducting a comparison of high-density *in situ* synthesized microarrays and massively-parallel signature sequencing (MPSS) data in the model plant, *Arabidopsis thaliana*. The MPSS data (available at http://mpss.udel.edu/at) and the microarray data have been compiled using the same RNA source material. In this review, we outline the experimental strategy that we are using, and present preliminary data and interpretations from the transcriptional profiles of *Arabidopsis* leaves and roots. The preliminary data indicate that the log ratio differences of transcripts between leaves and roots measured by microarray data are in better agreement with the MPSS data than the absolute intensities measured for individual microarrays hybridized to only one of the cRNA populations. The correlation was substantially improved by focusing on a subset of genes excluding those with very low expression levels; this selection may have removed noisy data. Future reports will incorporate more than 10 tissues that have been sampled by MPSS.


**Comparative and Functional Genomics**
*Comp Funct Genom* 2004; **5**: 245–252.
Published online in Wiley InterScience (www.interscience.wiley.com). **DOI:** 10.1002/cfg.397

**Conference Review**

### A comparison of global gene expression measurement technologies in *Arabidopsis thaliana*

Sean J. Coughlan[1]*, Vikas Agrawal[2] and Blake Meyers[2]
[1]*Agilent Technologies Inc., Little Falls Site, 2850 Centerville Road, Wilmington, DE 19808-1644, USA*
[2]*Department of Plant and Soil Sciences and Delaware Biotechnology Institute, University of Delaware, 15 Innovation Way, Newark, DE 19711, USA*

**Correspondence to: Sean J. Coughlan, Agilent Technologies Inc., Little Falls Site, 2850 Centerville Road, Wilmington, DE 19808-1644, USA. E-mail: sean_coughlan@agilent.com*

Received: 10 February 2004
Revised: 12 February 2004
Accepted: 12 February 2004

### Abstract

**Microarrays and tag-based transcriptional profiling technologies represent diverse but complementary data types. We are currently conducting a comparison of high-density *in situ* synthesized microarrays and massively-parallel signature sequencing (MPSS) data in the model plant, *Arabidopsis thaliana*. The MPSS data (available at http://mpss.udel.edu/at) and the microarray data have been compiled using the same RNA source material. In this review, we outline the experimental strategy that we are using, and present preliminary data and interpretations from the transcriptional profiles of *Arabidopsis* leaves and roots. The preliminary data indicate that the log ratio differences of transcripts between leaves and roots measured by microarray data are in better agreement with the MPSS data than the absolute intensities measured for individual microarrays hybridized to only one of the cRNA populations. The correlation was substantially improved by focusing on a subset of genes excluding those with very low expression levels; this selection may have removed noisy data. Future reports will incorporate more than 10 tissues that have been sampled by MPSS. Copyright © 2004 John Wiley & Sons, Ltd.**

**Keywords:** **gene expression; MPSS; microarrays; cross-platform comparisons**

### Introduction

The establishment of whole genome sequences and the parallel development of technologies such as high density DNA microarrays and tag-based gene expression systems have enabled 'global' measurements of gene expression. In the plant community, the genomic sequences of *Arabidopsis thaliana* [1] and rice [2,3] are complete or nearly complete. These sequences can serve as templates for the design of high-density oligonucleotide microarrays. Several microarray array platforms have been produced based on the *Arabidopsis* sequence, including first-generation arrays produced by a public consortium (the *Arabidopsis* Functional Genomics Consortium, or AFGC) [4] and a commercial array that included more than 8000 *Arabidopsis* genes produced by Affymetrix Inc. (Santa Clara, CA) [5]. The Affymetrix GeneChip arrays are comprised of sets of 25-base oligonucleotides synthesized *in situ* via a photolithographic process [6]. The most recent generation of commercially-produced *Arabidopsis* arrays include more than 21 000 genes. One such array is produced by Affymetrix. A second commercial array is produced by Agilent Technologies Inc. (Palo Alto, CA) and is based on the process of ink-jet 'printing' of 60-base probes [7]. Microarrays produce a comparative, or qualitative, measurement of gene expression, based on relative measures of dye intensities that correspond to the amount of target RNA that has hybridized to a specific probe. For an experimental condition, the relative signal intensity of each gene is measured against that of a control tissue.

Copyright © 2004 John Wiley & Sons, Ltd.

An alternative set of gene expression technologies provides absolute, quantitative measures of gene expression. These tag- or sequence-based technologies determine the expression level of a gene by counting the precise abundance of a specific transcript in a library. There are two widely used methods for quantitative measurements of gene expression, SAGE (Serial Analysis of Gene Expression) [8] and MPSS (Massively Parallel Signature Sequencing) [9]. Both of these technologies produce short (10–22 nucleotide) sequence tags that are derived from a defined position in the mRNA molecule. A significant advantage of the MPSS technology relative to SAGE is the large number of signatures (more than 1 000 000) that are rapidly generated for a given library. In addition, the tags produced by MPSS are longer than most SAGE tags, being 17 or 20 bases in length. The longer MPSS tags uniquely match the majority of genes in the *Arabidopsis* genome and permit specific identification of transcribed regions [10]. The approximate location of the polyadenylation site for each transcript is known because both SAGE and MPSS tags are derived from defined restriction sites in the 3′ end of a transcript.

Because microarrays and tag-based methods are now able to measure nearly all of the more than 29 000 genes that are annotated in the *Arabidopsis* genome, we are undertaking a comparison of the qualitative and quantitative measurements produced by these technologies. MPSS data are available for more than 10 *Arabidopsis* tissues or treatments [10; and **http://mpss.udel.edu/at**]. We used the same RNA that was sequenced by MPSS as a template for microarray analysis. The microarrays in use in our analysis are the Agilent *in situ* synthesized 'long oligo' arrays. The contents of this report, describing our initial results of this direct comparison, were presented at the 12th International Plant and Animal Genome conference in San Diego, California, on 13 January 2004.

### Platforms for global gene expression analysis

The *Arabidopsis* MPSS database currently contains 14 libraries, representing 11 distinct samples (Table 1); sample names in Table 1 that are followed by a '2' have been sequenced in duplicate with a variation on the MPSS technology (B. C. Meyers, unpublished). These samples represent different developmental stages (libraries 1–5 include callus, inflorescence, leaves, roots and silique) [11], a series of homeotic floral mutants (libraries 6–9 and 11 include *apetala1-10, apetala3-6, agamous* and a *superman/apetala1-10* double mutant) (H. Ghazal, H. Sakai and B. C. Meyers, unpublished) and chemical treatments (libraries 12 and 13, include 4 h and 52 h after salicylic acid treatment) (M. West, D. St. Clair and B. C. Meyers, unpublished). For this analysis, we are focusing on the 17-base signatures, rather than the 20-base signatures, because the shorter tags represent a less-biased set [10]. Each of these 17-base libraries contained more than 1.75 million signatures derived from four to eight sequencing runs, with an average of 2.64 million signatures per run. These signatures represented an average of 42 778 distinct signatures per library, of which on average 19 750 were present at levels considered significant and reliable. This depth approximates a genome-wide coverage of more than 99% of *Arabidopsis* genes [10]. The raw abundance of each signature in each library is normalized to a metric of 'transcripts per million' (TPM), which facilitates comparisons across libraries. To obtain an expression level for each gene, we summed the abundance of signatures that match to the sense-strand of the gene and are found only once in the *Arabidopsis* genome. By summing the signatures, all

**Table 1.** MPSS Libraries available for platform comparisons

| No | Library | Code | Total 17-base signatures | Total 20-base signatures |
|---|---|---|---|---|
| 1 | Callus | CAF | 1 959 539 | 1 637 407 |
| 2 | Inflorescence | INF | 1 774 306 | 1 455 847 |
| 3 | Leaves | LEF | 2 884 598 | 2 457 736 |
| 4 | Roots | ROF | 3 642 632 | 3 002 218 |
| 5 | Silique | SIF | 2 012 859 | 1 673 908 |
| 6 | *ap1-10* inflor. | AP1 | 2 964 724 | 2 558 992 |
| 7 | *ap3-6* inflor. | AP3 | 2 435 965 | 2 050 647 |
| 8 | *agamous* inflor. | AGM | 2 575 670 | 2 165 628 |
| 9 | Inflorescence–2 | INS | 2 890 894 | 2 516 138 |
| 10 | Roots–2 *sup/ap1-10* | ROS | 2 458 436 | 2 047 569 |
| 11 | Inflor. | SAP | 2 310 350 | 1 874 297 |
| 12 | SA 4 h–leaf | S04 | 3 006 975 | 2 626 946 |
| 13 | SA 52 h–leaf | S52 | 2 964 840 | 2 584 795 |
| 14 | Leaves–2 | LES | 3 109 385 | 2 752 425 |
| | Total | | 36 991 173 | 31 404 553 |

Table modified from [10].

Copyright © 2004 John Wiley & Sons, Ltd. 

alternatively-polyadenylated transcripts are considered together.

To generate comparable microarray data, aliquots of the same *Arabidopsis* RNA samples used for MPSS analysis serve as templates for labelled cRNA synthesis. This target material is being hybridized to three available high-density *Arabidopsis* microarrays. These are the Affymetrix ATH1 GeneChip (22 800 features, ~10 probes per gene of 25 nucleotides each), the Agilent 22K microarray (21 500 features, 60 nucleotide probes, one per gene, synthesized *in situ*) and a spotted long-oligo array built using the 26 000 element Operon/Qiagen *Arabidopsis* oligos (60 nucleotide probes, one per gene) produced at the University of Arizona [**http://www.ag.arizona.edu/microarray/**]. A total of 29 389 genes are listed in the most recent *Arabidopsis* annotation (TIGR version 4.0, June 2003) [12]. By way of comparison, we have estimated that MPSS should be able to detect 29 151 genes; more than 200 genes lack a *Dpn*II site that is required for detection with this technology. The goal of our experiments is to compare these three microarray platforms to MPSS to estimate the degree of correlation and agreement across these disparate technology platforms.

Because these microarray platforms are based on different annotations of the *Arabidopsis* genome and contain only a subset of the total annotated Arabidopsis genes, we investigated the number of genes represented on all four technology platforms. Gene lists for all four platforms were compared, using gene identifiers for which probe sets are available; this analysis demonstrated that there are 17 118 genes in common (Figure 1). This subset of genes will be most informative because expression data can be compared across the platforms. However, the presence of this common set of genes on the different arrays belies numerous potential differences in measurements, due to variability among manufacturers in the oligo length, position within a gene, and numbers of probes for each gene. We anticipate that some genes will be better measured by the probes on certain microarray platforms, and no single platform will accurately measure every gene. The process of correlating design features with expression data is likely to require substantial empirical data across many different designs. And as genomic annotations begin to incorporate information about splice variants and functional noncoding transcripts, it will be important for array manufacturers to agree on a set of standard template sequences, to ensure that probes with identical gene identifiers are measuring precisely the same transcripts.

At this time, we have generated data for the comparison of the Agilent *Arabidopsis* microarrays to the MPSS dataset. The Agilent arrays consist of 60-nucleotide oligomers fabricated *in situ* using a maskless, 'ink-jet' synthesis reaction [13]. We determined the reproducibility and the dynamic range of these arrays by hybridizing a single sample labelled with both Cy3 and Cy5 dyes; the results from the leaf sample are shown in Figure 2. The intensity values (Cy3 normalized vs. Cy5 normalized) were highly correlated ($r^2 > 0.98$), suggesting little experimental variation in the dye incorporation. The intensity data displayed a range of more than 3.5 orders of magnitude, from about 50 units (twice background) to more than 200 000 units. At the upper end of the dynamic range, we found that slightly more than 0.1% of the features were saturated. Saturation of the hybridization signal of high-abundance transcripts

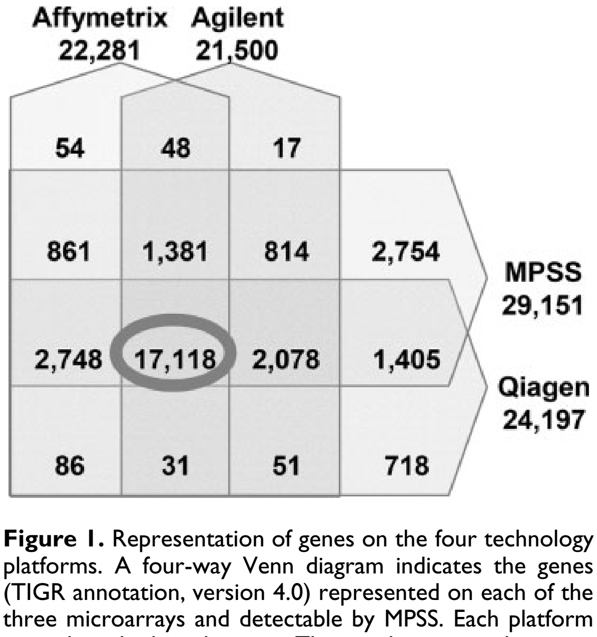

**Figure 1.** Representation of genes on the four technology platforms. A four-way Venn diagram indicates the genes (TIGR annotation, version 4.0) represented on each of the three microarrays and detectable by MPSS. Each platform is as described in the text. The numbers in each square indicate the genes shared across each set of microarray platforms. The number circled in grey indicates the subset of genes potentially detectable across all platforms

Copyright © 2004 John Wiley & Sons, Ltd. 

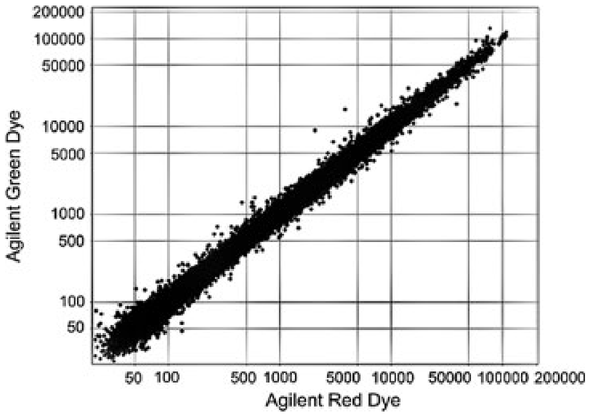

**Figure 2.** Reproducibility of the Agilent *Arabidopsis* microarray. A total of 400 ng *Arabidopsis* leaf RNA (MPSS library #3 source material) was used as template to generate cRNA using the Agilent low-input linear amplification kit. From the product of this amplification reaction, 750 ng cRNA (Cy3- or Cy5-labelled) was hybridized for 18 h at 65 °C to the Agilent 22K *Arabidopsis* microarray. The arrays were washed and scanned using the manufacturer's recommended conditions. The data shown are for two replicate arrays with dye-normalized intensities plotted on a logarithmic scale results in underestimation of expression, and biases estimates of expression for the most abundant transcripts. This low level of saturation is important for the cross-platform comparison. Because MPSS is a 'digital' count of abundance, it should have a broad linear range with no saturation at the upper abundance level; any probe sets that are saturated on the microarrays would correlate poorly with the unsaturated MPSS data.

### Comparison of gene expression data derived from MPSS and microarrays

First, we assessed the ability of the Agilent microarray to detect differential expression among two distinct tissues, leaf and root. The basis for this analysis was to generate a baseline for later comparison to the MPSS data. The RNA from leaf and root was used as template for cRNA synthesis and labelling; we used four arrays, with two replicates each of leaf (Cy3-labelled) and root (Cy5-labelled), followed by the corresponding dye-swap in which the leaf is labelled with Cy5 and the root with Cy3 (Figure 3). This represents a technical replication of each dye-swap and, because both samples were present on each array, we had a total of four replications. Technical replications, in this case represented by separate labelling reactions and arrays run for the same RNA sample,

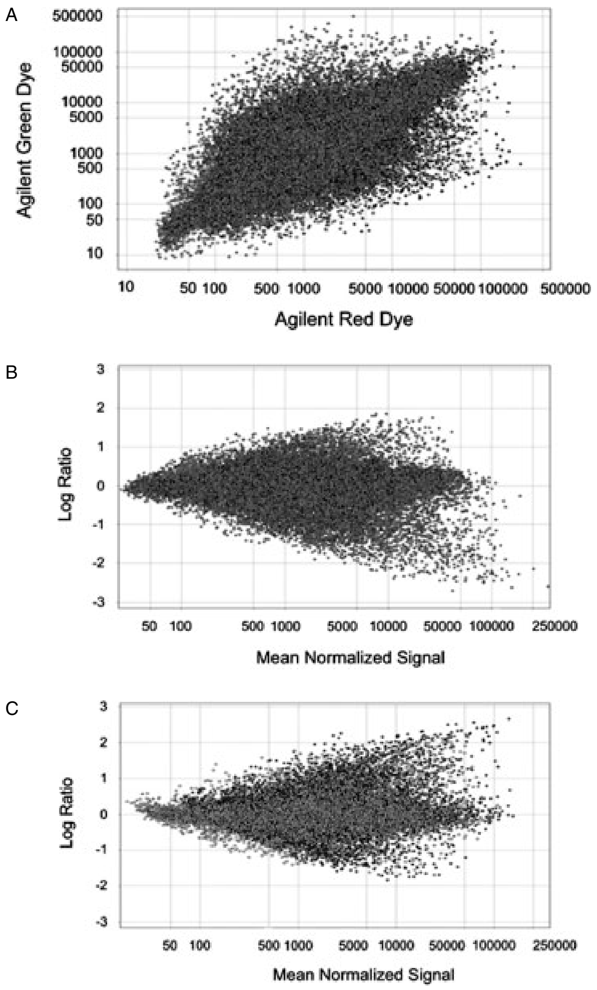

**Figure 3.** Comparison of microarray data for leaf and root samples. *Arabidopsis* leaf (MPSS library #3) and *Arabidopsis* root (MPSS library #2) were compared. As in Figure 2, 400 ng input RNA was amplified and 750 ng labelled cRNA was hybridized to Agilent 22K *Arabidopsis* microarrays. Dye-swaps were performed, and replicates were obtained for each dye configuration (leaf Cy3/root Cy5 for one array, and leaf Cy5/root Cy3 for the second array). The data shown are averaged across four microarrays, representing two arrays for each dye-swap polarity. (A) Scatter graph of dye-normalized intensities on a logarithmic scale. (B) Graph of log ratio (intensity) linear scale against mean normalized intensity (logarithmic scale) showing positive (+1) polarity (leaf Cy3/root Cy5 only). (C) Graph of log ratio (intensity) linear scale against mean normalized intensity [logarithmic scale showing negative (−1) polarity (leaf Cy5/root Cy3 only)]

Copyright © 2004 John Wiley & Sons, Ltd.

are essential to minimize and quantify variance in the array experiments [14]. In an ideal experiment, we would also use biological replications, or RNA samples extracted from separate, but identically-treated biological materials. However, the cost of MPSS is prohibitive for biological replicates, and in this case the point of our analysis is to test the correlations of the technology platforms and not necessarily extract the biological information from this analysis. The comparison of RNA from two different plant organs identifies differences in gene expression profiles for at least 1000 transcripts that were upregulated 10-fold in leaf compared to root, with more than 500 transcripts upregulated by 10-fold in root compared to the leaf (Figure 3). The log ratio data corresponding to the differences in gene expression between the leaf and root samples were later compared to corresponding differences in MPSS data.

Next, for the leaf library, we compared the signal intensity for genes represented on the Agilent microarray with the corresponding MPSS data (Figure 4). For each gene, the total 'dye-normalized' intensity was calculated by summing the total signal intensity for both channels and dividing by the total signal intensity observed on the microarray; this value was then averaged across replicates. A plot of the dye-normalized intensity against the TPM values for corresponding genes demonstrates a weak correlation between the two overall datasets ($r^2 = 0.43$) (Figure 4). The horizontal bands of points that are parallel to the $x$ axis result from the log-scale plot and the discrete values for expression (in 'TPM') used in the MPSS data. It is also apparent from this plot that there are numerous transcripts (approximately 500) for which hybridization data were detected by the microarray, but for which no or very little

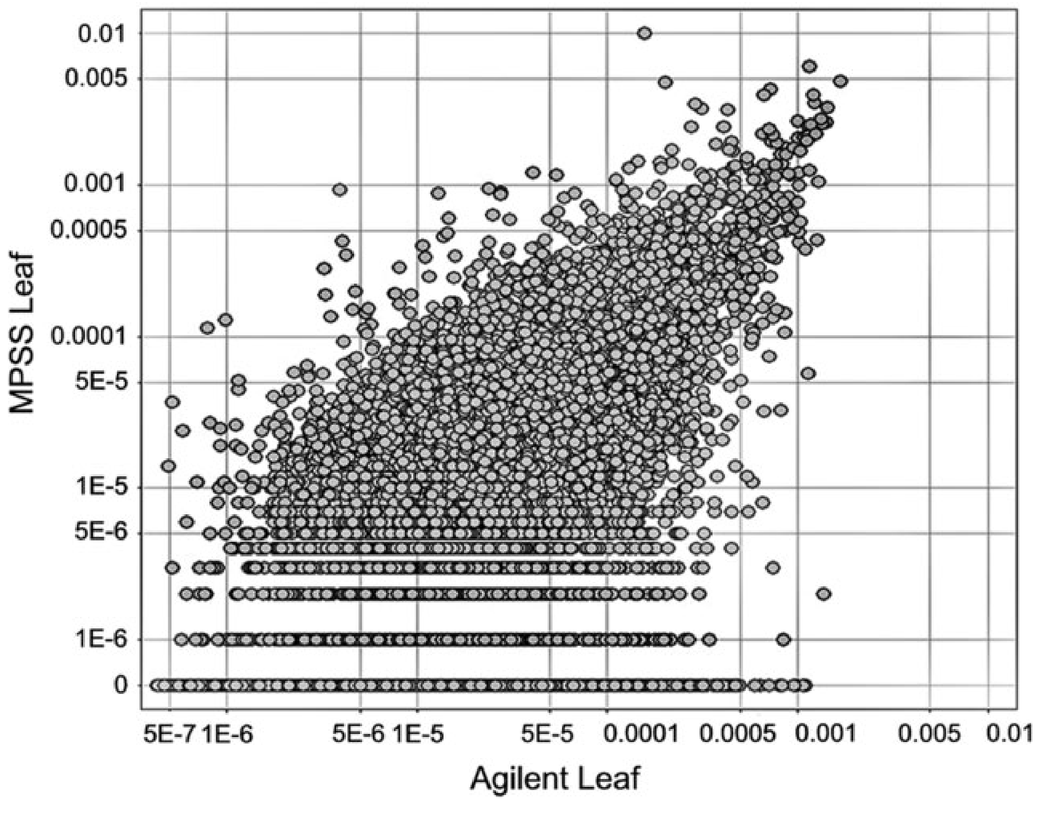

**Figure 4.** Comparison of MPSS signature abundance with microarray feature signal intensity. The data for the leaf sample (MPSS library #3) are shown as a scatter plot on a logarithmic scale. MPSS signature abundances in TPM were compared to the total dye-normalized signal intensity. For each gene, the sum of the raw signal intensity for the Cy3 and Cy5 channels was divided by the sum over all genes on the array of the raw signal intensities for the Cy3 and Cy5 channels

Copyright © 2004 John Wiley & Sons, Ltd.

expression data were found in the MPSS analysis (Figure 4).

There are at least two systematic biases that are known to occur in the MPSS data that could explain the poor correlation at the lowest abundance levels for the MPSS and microarray expression data of the leaf sample. A small number of *Arabidopsis* genes (less than 300) are known to lack a suitable *Dpn*II site, a restriction site required for detection by MPSS [10]. A second and more significant bias results from the ∼7.7% of signatures that are underrepresented in the MPSS expression data, due to 'bad words' that are poorly sequenced by the technology. Because the association with genes of the underrepresented signatures is essentially random, and because multiple expressed signatures may be associated with a single gene (primarily due to alternative polyadenylation), this bias may produce noise that reduces the MPSS-measured expression level of a given gene, without lowering it all the way to zero TPM. We believe that this second bias is responsible for the substantial number of genes indicated in Figure 4 that show lower expression levels in the MPSS data compared to the microarray data. To compensate for the large number of genes with expression levels less than 10 TPM by MPSS, we recalculated the correlation of the MPSS and microarray data using only the more abundantly expressed genes; in this case, the correlation was much stronger ($r^2 = 0.75$).

Finally, we compared the differentially expressed gene expression data obtained by microarray analysis with the corresponding MPSS data (Figure 5). Transcripts which were upregulated in the leaf relative to the root are indicated as the set that have a negative log ratio (Figure 3B). This unfiltered data, when analysed by linear regression, showed a moderate correlation ($y = 1.3546x + 0.0442$; $r^2 = 0.53$). For simplicity, the dye swap data (two arrays with Cy5-labelled leaf RNA and Cy3-labelled root

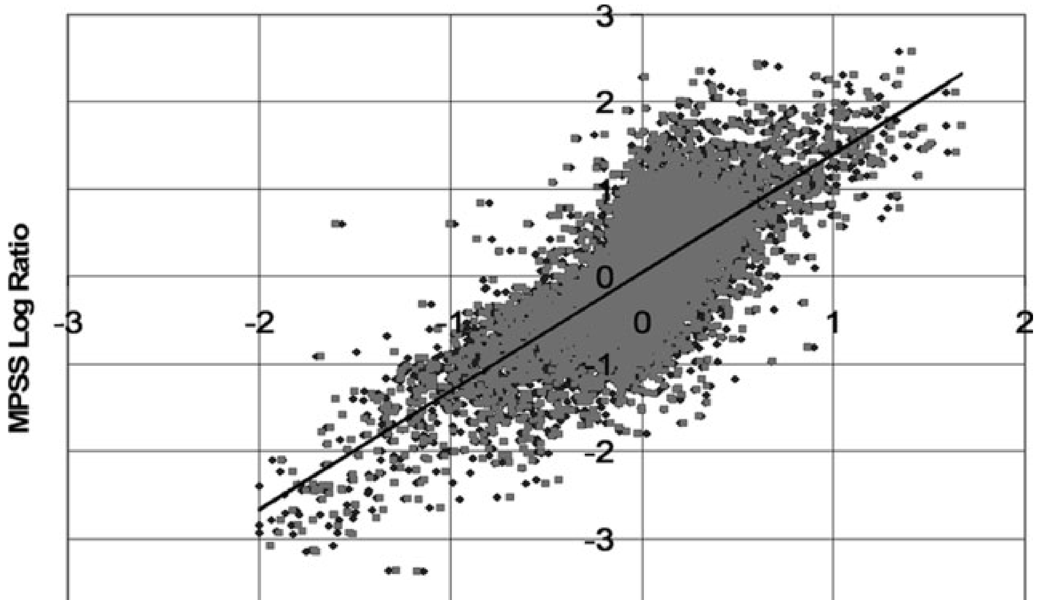

**Figure 5.** Comparison of MPSS signature abundance differences with microarray log-ratio differences for leaf and root samples. The differences between the leaf and root samples were determined for the MPSS data and for the Agilent 22K microarray. The magnitude of differential expression detected by the two technology platforms is indicated by in the plot. On the *x* axis, the log ratio for the Agilent arrays calculated as the log of the ratio of leaf (Cy3-labelled) over root (Cy5-labelled) fluorescent intensities which had been corrected both for non-specific background intensities, and for the different labelling efficiencies, spectral emission and adsorption coefficients of the Cy3 and Cy5 (i.e. these are background-subtracted and dye-normalized intensities). On the *y* axis, the MPSS data were used to calculate the log of the ratio of the root abundance (in TPM) over the leaf abundance (in TPM). The data shown is for two replicate arrays

Copyright © 2004 John Wiley & Sons, Ltd. 

RNA) is not included in this analysis, but these data were essentially identical (data not shown). The log ratio comparisons of the differentially expressed genes between leaf and root (Figure 5) produced a better correlation than that observed when the absolute microarray signal intensities for either tissue alone were compared to MPSS transcript abundance (e.g. Figure 4). When we applied the same filters used in the differentially expressed data to exclude genes with low expression levels (<10 TPM) in both leaf and root, the correlation increased substantially ($r^2 = 0.85$). These data demonstrate well-correlated measurements of comparative abundance between samples with both microarrays and MPSS for a subset of genes. Based on this finding and the single-tissue comparison of MPSS and microarray data described above (Figure 4), the creation of subsets of genes for which the measurements of expression have been empirically validated in different ways may improve the correlations between technology platforms.

The next stage of the analysis will incorporate data from at least 10 of the MPSS libraries, compared with the three microarray platforms. In collaboration with laboratories at the University of California at Davis (D. St. Clair and R.W. Michelmore), we are currently generating data from the Affymetrix ATH1 GeneChip, including technical replicates. The University of Arizona microarray facility (D. Galbraith) is generating data from the spotted long-oligo microarrays. When the dataset is complete, additional statistical tools will be employed to assess the correlations among the different technology platforms.

The statistical approaches that we are using for the cross-platform comparisons are similar to those described in Tan *et al.* [15]. As these authors indicate, one difficulty in working with disparate data types is that gene expression measurements are reported in units unique to each platform. Therefore, to directly compare data between platforms, we will need to convert these measurements to a single common scale, based on the original fluorescent signals in the raw microarray data, and this will need to be compatible with the MPSS units normalized to 'transcripts per million'. As described by Tan *et al.* [15], one approach is to rescale the data by application of a Z-transformation, so that the mean and variance (mean = 0 and standard deviation = 1) of the signals are equivalent for *Arabidopsis* genes shared across the platforms, permitting direct comparisons of signal distributions and error levels across technologies. To compute correlations of the signal across platforms, we are computing Pearson linear correlation coefficients and Spearman rank-order correlation coefficients for shared genes in each pair of platforms, using expression measurements that are computed as the mean of the replicate arrays. An appropriate statistical approach to isolate and identify sources of variation are the ANOVA-based tools that have been used in some published studies of microarray data [16]. As a result of sequence-specific biases in the MPSS data, approximately 9% of all signatures are underrepresented in the MPSS dataset [11].

Several comparisons among microarray platforms have suggested that we are likely to find a poor correlation among different platforms. For example, the identical human RNA samples used by Tan *et al.* [15] on the Affymetrix (25-mer), Agilent (60-mer) and Amersham (30-mer) microarray platforms produced variable results across the platforms. In their study, considerable variation was observed among the subsets of genes showing significant changes in expression, and the correlations in gene expression levels across the different platforms were quite modest (Pearson's correlation coefficient, average 0.53, range 0.48–0.60). In addition, although many of the genes present on each of these human microarray platforms were the same (as with *Arabidopsis*), the differentially expressed genes identified by each technology did not substantially overlap. Other studies have found a poor correlation between spotted cDNA microarrays and Affymetrix GeneChip arrays [17,18], perhaps because these are significantly different types of array platforms. Each of these studies suggests that the conclusions derived from a microarray analysis are dependent upon the type of platform used for the experiment.

### Conclusions and prospects

Our observations of moderate or low correlations among the two data types agree with previous studies that compared SAGE data to microarray data for other organisms [19–21]. By focusing on genes that are most reliably detected in both microarray

Copyright © 2004 John Wiley & Sons, Ltd.

and MPSS analyses, we are likely to improve the correlations that we have reported here. One of our goals will be to define these subsets of genes using empirically-derived microarray and MPSS data. Because the limit of detection for some commercial microarrays is close to ~1/100 000 transcripts, expression and changes in gene expression near this level are difficult to detect with statistical significance [20]. Therefore, our comparison is likely to focus on moderately expressed genes observed at levels greater than 10 TPM in the MPSS database, because these data may be more robust than the weakly expressed genes.

The increasing availability of high-quality genomic sequence and annotation data will provide new resources for the construction of platforms for whole-genome transcriptional analysis. The data produced by hybridization-based platforms like microarrays may not be directly comparable with those produced by sequence-tag-based platforms like SAGE or MPSS. However, these platforms are complementary and each type of platform has advantages and disadvantages. SAGE and MPSS represent 'open' platforms, for which the measured transcripts are not pre-selected; these data can be used to find novel transcripts that may be missed by computational and predictive annotation systems. In contrast, microarrays provide a relatively inexpensive means for analysing expression and identifying differentially regulated transcripts under a broad range of conditions. The application of *both* tag-based expression measurements and microarrays to the growing number of complex eukaryotic genomes that are being sequenced will enable transcript identification and quantification to proceed at a greatly increased rate compared to the first generation of sequenced genomes.

### Acknowledgements

We would like to thank the groups of David Galbraith (University of Arizona) and Richard Michelmore and Dina St Clair (University of California at Davis) for ongoing collaborations. The MPSS data was generated with support from NSF Plant Genome Research Award No. 0110528 (to B.C.M.).

### References

1. *Arabidopsis* Genome Initiative. 2000. Analysis of the genome sequence of the flowering plant *Arabidopsis thaliana*. *Nature* **408**: 796–815.
2. Goff SA, Ricke D, Lan TH, *et al.* 2002. A draft sequence of the rice genome (*Oryza sativa* L. ssp. *japonica*). *Science* **296**: 92–100.
3. Yu J, Hu S, Wang J, *et al.* 2002. A draft sequence of the rice genome (*Oryza sativa* L. ssp. *indica*). *Science* **296**: 79–92.
4. Wisman E, Ohlrogge J. 2000. *Arabidopsis* microarray service facilities. *Plant Physiol* **124**: 1468–1471.
5. Zhu T, Wang X. 2000. Large-scale profiling of the *Arabidopsis* transcriptome. *Plant Physiol* **124**: 1472–1476.
6. Lockhart DJ, Dong H, Byrne MC, *et al.* 1996. Expression monitoring by hybridization to high-density oligonucleotide arrays. *Nature Biotechnol* **14**: 1675–1680.
7. Hughes TR, Mao M, Jones AR, *et al.* 2001. Expression profiling using microarrays fabricated by an ink-jet oligonucleotide synthesizer. *Nature Biotechnol* **19**: 342–347.
8. Velculescu VE, Zhang L, Vogelstein B, Kinzler KW. 1995. Serial analysis of gene expression. *Science* **270**: 484–487.
9. Brenner S, Johnson M, Bridgham J, *et al.* 2000. Gene expression analysis by massively parallel signature sequencing (MPSS) on microbead arrays. *Nature Biotechnol* **18**: 630–634.
10. Meyers BC, Tej SS, Vu TH, *et al.* 2004. The use of MPSS for whole-genome transcriptional analysis in *Arabidopsis*. *Genome Res* (submitted).
11. Meyers BC, Vu TH, Tej SS, *et al.* 2004. Analysis of the *Arabidopsis* transcriptome by massively parallel signature sequencing. *Nature Genetics* (submitted).
12. Wortman JR, Haas BJ, Hannick LI, *et al.* 2003. Annotation of the *Arabidopsis* genome. *Plant Physiol* **132**: 461–468.
13. Shoemaker DD, Schadt EE, Armour CD, *et al.* 2001. Experimental annotation of the human genome using microarray technology. *Nature* **409**: 922–927.
14. Lee ML, Kuo FC, Whitmore GA. Sklar J. 2000. Importance of replication in microarray gene expression studies: statistical methods and evidence from repetitive cDNA hybridizations. *Proc Natl Acad Sci USA* **97**: 9834–9839.
15. Tan PK, Downey TJ, Spitznagel EL Jr, *et al.* 2003. Evaluation of gene expression measurements from commercial microarray platforms. *Nucleic Acids Res* **31**: 5676–5684.
16. Jin W, Riley RM, Wolfinger RD, *et al.* 2001. The contributions of sex, genotype and age to transcriptional variance in *Drosophila melanogaster*. *Nature Genet* **29**: 389–395.
17. Kuo WP, Jenssen T-K, Butte AJ, *et al.* 2002. Analysis of matched mRNA measurements from two different microarray technologies. *Bioinformatics* **18**: 405–412.
18. Yuen T, Wurmbach E, Pfeffer RL, *et al.* 2002. Accuracy and calibration of commercial oligonucleotide and custom cDNA microarrays. *Nucleic Acids Res* **30**: e48.
19. Iacobuzio-Donahue CA, Ashfaq R, Maitra A, *et al.* 2003. Highly expressed genes in pancreatic ductal adenocarcinomas: a comprehensive characterization and comparison of the transcription profiles obtained from three major technologies. *Cancer Res* **63**: 8614–8622.
20. Ishii M, Hashimoto S, Tsutsumi S, *et al.* 2000. Direct comparison of GeneChip and SAGE on the quantitative accuracy in transcript profiling analysis. *Genomics* **68**: 136–143.
21. Kim HL. 2003. Comparison of oligonucleotide-microarray and serial analysis of gene expression (SAGE) in transcript profiling analysis of megakaryocytes derived from $CD34^+$ cells. *Exp Mol Med* **35**: 460–466.

Copyright © 2004 John Wiley & Sons, Ltd. 
